# RNAenrich: a web server for non-coding RNA enrichment

**DOI:** 10.1093/bioinformatics/btad421

**Published:** 2023-07-03

**Authors:** Song Zhang, Kuerbannisha Amahong, Yintao Zhang, Xiaoping Hu, Shijie Huang, Mingkun Lu, Zhenyu Zeng, Zhaorong Li, Bing Zhang, Yunqing Qiu, Haibin Dai, Jianqing Gao, Feng Zhu

**Affiliations:** Center for Clinical Pharmacy, Cancer Center, Department of Pharmacy, Zhejiang Provincial People’s Hospital, Affiliated People's Hospital, Hangzhou Medical College, Hangzhou, Zhejiang 310014, China; College of Pharmaceutical Sciences, The Second Affiliated Hospital, Zhejiang University School of Medicine, Zhejiang University, Hangzhou, Zhejiang 310058, China; College of Pharmaceutical Sciences, The Second Affiliated Hospital, Zhejiang University School of Medicine, Zhejiang University, Hangzhou, Zhejiang 310058, China; College of Pharmaceutical Sciences, The Second Affiliated Hospital, Zhejiang University School of Medicine, Zhejiang University, Hangzhou, Zhejiang 310058, China; Center for Clinical Pharmacy, Cancer Center, Department of Pharmacy, Zhejiang Provincial People’s Hospital, Affiliated People's Hospital, Hangzhou Medical College, Hangzhou, Zhejiang 310014, China; College of Pharmaceutical Sciences, The Second Affiliated Hospital, Zhejiang University School of Medicine, Zhejiang University, Hangzhou, Zhejiang 310058, China; College of Pharmaceutical Sciences, The Second Affiliated Hospital, Zhejiang University School of Medicine, Zhejiang University, Hangzhou, Zhejiang 310058, China; Innovation Institute for Artificial Intelligence in Medicine of Zhejiang University, Alibaba-Zhejiang University Joint Research Center of Future Digital Healthcare, Hangzhou, Zhejiang 330110, China; Innovation Institute for Artificial Intelligence in Medicine of Zhejiang University, Alibaba-Zhejiang University Joint Research Center of Future Digital Healthcare, Hangzhou, Zhejiang 330110, China; Innovation Institute for Artificial Intelligence in Medicine of Zhejiang University, Alibaba-Zhejiang University Joint Research Center of Future Digital Healthcare, Hangzhou, Zhejiang 330110, China; State Key Laboratory for Diagnosis and Treatment of Infectious Disease, Collaborative Innovation Center for Diagnosis and Treatment of Infectious Diseases, Zhejiang Provincial Key Laboratory for Drug Clinical Research and Evaluation, The First Affiliated Hospital, Zhejiang University, Hangzhou, Zhejiang 310000, China; College of Pharmaceutical Sciences, The Second Affiliated Hospital, Zhejiang University School of Medicine, Zhejiang University, Hangzhou, Zhejiang 310058, China; College of Pharmaceutical Sciences, The Second Affiliated Hospital, Zhejiang University School of Medicine, Zhejiang University, Hangzhou, Zhejiang 310058, China; Westlake Laboratory of Life Sciences and Biomedicine, Hangzhou, Zhejiang 310030, China; Center for Clinical Pharmacy, Cancer Center, Department of Pharmacy, Zhejiang Provincial People’s Hospital, Affiliated People's Hospital, Hangzhou Medical College, Hangzhou, Zhejiang 310014, China; College of Pharmaceutical Sciences, The Second Affiliated Hospital, Zhejiang University School of Medicine, Zhejiang University, Hangzhou, Zhejiang 310058, China

## Abstract

**Motivation:**

With the rapid advances of RNA sequencing and microarray technologies in non-coding RNA (ncRNA) research, functional tools that perform enrichment analysis for ncRNAs are needed. On the one hand, because of the rapidly growing interest in circRNAs, snoRNAs, and piRNAs, it is essential to develop tools for enrichment analysis for these newly emerged ncRNAs. On the other hand, due to the key role of ncRNAs’ interacting target in the determination of their function, the interactions between ncRNA and its corresponding target should be fully considered in functional enrichment. Based on the ncRNA–mRNA/protein-function strategy, some tools have been developed to functionally analyze a single type of ncRNA (the majority focuses on miRNA); in addition, some tools adopt predicted target data and lead to only low-confidence results.

**Results:**

Herein, an online tool named RNAenrich was developed to enable the comprehensive and accurate enrichment analysis of ncRNAs. It is unique in (i) realizing the enrichment analysis for various RNA types in humans and mice, such as miRNA, lncRNA, circRNA, snoRNA, piRNA, and mRNA; (ii) extending the analysis by introducing millions of experimentally validated data of RNA–target interactions as a built-in database; and (iii) providing a comprehensive interacting network among various ncRNAs and targets to facilitate the mechanistic study of ncRNA function. Importantly, RNAenrich led to a more comprehensive and accurate enrichment analysis in a COVID-19-related miRNA case, which was largely attributed to its coverage of comprehensive ncRNA–target interactions.

**Availability and implementation:**

RNAenrich is now freely accessible at https://idrblab.org/rnaenr/.

## Graphical Abstract

**Graphical Abstract:**
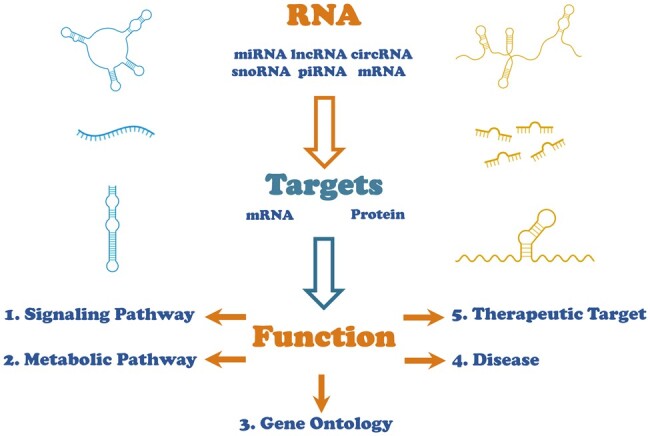

## 1 Introduction

Over the past quarter-century, non-coding RNAs (ncRNAs) have guided a prevalent trend in biomedical and life science research, and interest in ncRNAs and associated fields is continually expanding (Lee *et al.* 2020, Vidovic *et al.* 2020, Venkatesh *et al.* 2021, Yoo *et al.* 2021a). With the increasing amount of in depth ncRNA research, several kinds of regulatory ncRNAs, including microRNA (miRNA) (Gebert and MacRae 2019), long non-coding RNA (lncRNA) (Elling *et al.* 2018, Statello *et al.* 2021, Vollmers *et al.* 2021), circular RNA (circRNA) (Zeng *et al.* 2017, Kristensen *et al.* 2019, Jia *et al.* 2020), small nucleolar RNA (snoRNA) (Liang *et al.* 2019), and piwi-interacting RNA (piRNA) (Ozata *et al.* 2019), have received enormous attention from researchers due to their capacities to orchestrate gene expression (Chen *et al.* 2017, 2019b, Wang *et al.* 2021a). Uncovering their molecular mechanism during cellular processes is crucial for solving physiological and pathophysiological problems (Beermann *et al.* 2016, Goodall and Wickramasinghe 2021). Currently, the theoretical system of ncRNA regulation has been well established based on the cumulation of millions of published studies (Zeng *et al.* 2017, Elling *et al.* 2018, Gebert and MacRae 2019, Kristensen *et al.* 2019, Jia *et al.* 2020, Statello *et al.* 2021, Vollmers *et al.* 2021). For example, miRNA can bind to specific mRNAs to influence their stability or translation (Pillman *et al.* 2018, Gebert and MacRae 2019); lncRNA and circRNA are frequently involved in the process of sustaining mRNA and protein stability via interactions, and can also absorb miRNAs to deprive their original function (Kristensen *et al.* 2019, Statello *et al.* 2021); snoRNA modulates gene expression by controlling mRNA processing (Liang *et al.* 2019); and piRNA induces gene silencing in the formation of RNA–protein complexes with piwi-subfamily Argonaute proteins (Ozata *et al.* 2019). Therefore, a growing number of databases have been developed to accommodate the collection of ncRNA-related interactions [e.g. miRNA–mRNA (Yang *et al.* 2020, Huang *et al.* 2022) and lncRNA–miRNA (Li *et al.* 2014, Cruickshank *et al.* 2021)], as well as other associations [e.g. ncRNA–disease (Tang *et al.* 2018, Ning *et al.* 2021), and ncRNA-pathway (Kehl *et al.* 2020)].

Enrichment analysis is frequently applied to describe the function (involved-disease, pathway, cell process, etc.) of a gene set during the process of disease development and reveal the molecular mechanism of diverse diseases (Delorey *et al.* 2021). With the development of ncRNA-related association databases, some computational tools that focus on the enrichment of ncRNAs have been well constructed (Wu and Watson 2009, Hsu *et al.* 2011, Vlachos *et al.* 2015, Li *et al.* 2018, Kern *et al.* 2020, Chen *et al.* 2021a, Olgun *et al.* 2021), among them, TAM (Li *et al.* 2018) and miEAA (Kern *et al.* 2020) carry out an enrichment analysis for miRNA, and LncSEA (Chen *et al.* 2021a) is for lncRNA. Currently, the available enrichment strategies for ncRNA include (i) mapping a ncRNA with a function (disease or pathway) according to the literature or database (Li *et al.* 2018, Kern *et al.* 2020) and (ii) matching a ncRNA with a target mRNA/protein according to the literature or prediction data and then matching the protein with a term according to a well-established knowledge hierarchy (Kern *et al.* 2021). As reported, the former fails to cover the effect of diverse ncRNA interactions; therefore, this strategy may catch less valuable information (Li *et al.* 2018, Kern *et al.* 2020). Meanwhile, the predicted data of the latter may reduce the accuracy of the enrichment results (Huntley *et al.* 2018, Kern *et al.* 2021). In other words, due to the increasing interest in circRNAs, snoRNAs and piRNAs, it is essential to enable enrichment analysis of these newly identified ncRNAs. Moreover, since the function of ncRNAs depends heavily on their interacting target, the interactions between ncRNA and the corresponding target should be fully considered in functional enrichment (Kern *et al.* 2021). However, little tool that enables these valuable functions has yet been available for diverse ncRNAs.

In this study, a novel online tool named RNAenrich was developed to enable comprehensive functional enrichment analysis of diverse human and mouse ncRNAs. First, the interaction data between ncRNAs (circRNA, lncRNA, miRNA, piRNA, snoRNA, etc.) and their corresponding targets were collected using a systematic literature review in PubMed and various existing databases, which resulted in ∼1.87 million experimentally validated interactions. Second, enrichment analysis was realized by supporting a variety of functional categories, such as signaling pathway, metabolic pathway, Gene Ontology, disease, and therapeutic target. To the best of our knowledge, this tool is unique in that (i) it provides enrichment analysis for the most diverse types of ncRNA (not only lncRNA & miRNA, but also circRNA, piRNA & snoRNA) compared with existing tools; (ii) it extends the analysis by introducing millions of experiment-validated ncRNA–target interactions as a built-in database; and (iii) it provides an interacting network among various RNAs and different targets to facilitate the mechanistic study of ncRNA function. RNAenrich is now freely accessible without any login requirement at https://idrblab.org/rnaenr/.

## 2 Materials and methods

### 2.1 The functional enrichment strategy adopted in RNAenrich

Currently, the following two types of enrichment strategies for ncRNA sets are commonly used: putting an ncRNA into a disease, pathway, function, tissue location, etc., or matching an ncRNA with target functional mRNA/protein and then mapping the mRNA with various given terms. To ensure the diversity and reliability of the analysis, in this study, the second strategy was adopted. Therefore, the strategy of this study is matching an ncRNA with a target mRNA/protein according to experimentally validated data and then matching the protein with function according to well-established knowledge hierarchy, in which experimentally validated data ensure the accuracy of the enrichment results. Besides, the experimentally validated ncRNA interaction data were included high-confidence (low-throughput experimental data) and low-confidence (high-throughput experimental data) interactions. Users can choose confidence level according to their preferences. The tool has been developed so that an RNA list by RNA sequencing can be enriched to analyze associated functions, such as pathways and diseases.

RNAenrich can enrich five types of ncRNAs to analyze their function, including miRNAs, lncRNAs, circRNAs, snoRNAs, and piRNAs. All these ncRNAs generally interact with specific mRNAs or proteins and control their expression or activity, ultimately affecting the protein-induced signaling pathway. The following summary was well concluded from millions of studies: (i) miRNA generally interacts with specific mRNA and affects its stability and translation; (ii) lncRNA and circRNA can interact with miRNA to deprive its original functions and can also interact with mRNA and protein to affect their expression or activity; (iii) snoRNA regulates gene expression by controlling mRNA processing; and (iv) piRNA induces gene silencing by forming RNA–protein complexes with piwi-subfamily Argonaute proteins. In short, the functions of these five types of ncRNA heavily rely on interacting mRNAs or proteins. Therefore, the first step for RNAenrich analysis was matching the target mRNA/protein of the query ncRNA; all ncRNA–mRNA/protein interaction data were from diverse databases. After obtaining the targets of query ncRNA, the second step was carrying out an enrichment analysis via traditional enrichment databases, such as KEGG and Gene Ontology.

### 2.2 The ncRNA–target interaction data in RNAenrich

A variety of databases were included to determine the experimentally validated interactions, such as the miRNA–mRNA interactions from miRTarBase (Huang *et al.* 2022), miRecords (Xiao *et al.* 2009), miRSponge (Wang *et al.* 2015), OncomiRDB (Wang *et al.* 2014), Gene Ontology (Huntley *et al.* 2016, Gene Ontology Consortium 2021), IntAct (Del Toro *et al.* 2022), StarBase (Li *et al.* 2014), and TarBase (Karagkouni *et al.* 2018); lncRNA–mRNA, lncRNA–protein, and lncRNA–miRNA interactions in LncTarD (Zhao *et al.* 2020), LncACTdb (Wang *et al.* 2022b), LncRNA2Target (Cheng *et al.* 2019), and StarBase (Li *et al.* 2014); circRNA–mRNA, circRNA–protein, and circRNA–miRNA interactions circRNADisease (Zhao *et al.* 2018), StarBase (Li *et al.* 2014); snoRNA–mRNA and snoRNA–miRNA in SnoDB (Bouchard-Bourelle *et al.* 2020), StarBase (Li *et al.* 2014); and piRNA–mRNA and piRNA–protein in piRBase (Wang *et al.* 2022a). Among these, all interactions were recorded as strong- or weak-validation based on the selection of coefficient level. Overall, a total of 1.87 million ncRNA–target interaction data were collected and included in RNAenrich. These data are rich resources that are waiting for machine-learning tools (Chen *et al.* 2018b, Li *et al.* 2020, Meyer *et al.* 2020, Liu *et al.* 2021, Wang *et al.* 2021b, Hu *et al.* 2022a, Xia *et al.* 2022a, b, Li *et al.* 2023) to analyze, especially feature selection (Chen *et al.* 2018c, 2020, 2021b, Hu *et al.* 2021, 2022b, Too *et al.* 2022, Zhang *et al.* 2023) methods have great potential in this scenario.

### 2.3 The diverse functions that can be enriched in RNAenrich

Moreover, five popular databases were used in RNAenrich to facilitate the functional enrichment, including the KEGG (Kanehisa *et al.* 2021) and Reactome (Gillespie *et al.* 2022), which provided protein-directed signaling pathways that the ncRNA participate in; the SMPDB (Jewison *et al.* 2014), which offered metabolite-based pathways or reactions that ncRNAs regulate; Gene Ontology (Gene Ontology Consortium 2021), which contributed descriptions of the functional role of ncRNAs, their contribution to biological processes and location in the cell (especially, direct ncRNA biological process, cellular component, and molecular function annotations of Gene Ontology database are also included in built-in database of RNAenrich for enrichment analysis); TTD (Zhou *et al.* 2022) and KEGG (Kanehisa *et al.* 2021), which described the RNA-mediated occurrence and development of disease indication; and TTD (Zhou *et al.* 2022), which illustrated the therapeutic targets that ncRNAs regulate. Such diverse functional data can significantly enhance the capacity of ncRNA enrichment.

### 2.4 Server implementation details and required format of input

RNAenrich is deployed on a web server running Cent OS Linux v7.4.1708, Apache HTTP web server v2.4.6, and Apache Tomcat servlet container. Its web interface was developed by R v3.4.1 and Shiny v0.13.1 running on Shiny-server v1.4.1.759. Various R packages were utilized in the background processes. RNAenrich can be readily accessed by all users with no login requirement, and by diverse and popular web browsers, including Google Chrome, Mozilla Firefox, Safari, and Internet Explorer. The input is the ncRNA/gene set, which can be selected from the ncRNA/gene list of RNA sequencing/microarray data or the ncRNA/gene list of prediction data. To enhance the tolerance of RNAenrich analysis, RNAenrich allows diverse ID types as input RNA format for each RNA type. Five types of ncRNA (miRNA, lncRNA, circRNA, snoRNA, and piRNA) and mRNA lists can be analyzed in RNAenrich. RNAenrich allows users to select different ID lists (Table 1). RNAcentral is an authoritative and comprehensive ncRNA database in which each ncRNA is endowed with a unique ID (RNAcentral Consortium 2021). Therefore, all RNAs in RNAenrich are mapped to an RNAcentral ID if it is applicable in the RNAcentral database (RNAcentral does not include circRNAs and their corresponding information). In summary, six miRNA inputs can be accepted in RNAenrich, including RNAcentral ID, miRBase ID (mature), miRBase ID (stem-loop), Official Symbol, Gene ID, and miRNA name; five lncRNA inputs are accepted, including RNAcentral ID, Official Symbol, Gene ID, Ensembl ID, and lncRNA name; circRNA ID and circRNA name can be acceptable input format; five snoRNA inputs can be accepted, including RNAcentral ID, Official Symbol, Gene ID, Ensembl ID, and snoRNA name; RNAcentral ID and piRNA name can be input formats for mouse data; mRNA analysis allows five input formats, including Gene ID, Official Symbol, UniProt ID, Ensembl ID, and RefSeq ID. In addition, RNAenrich supports one type of RNA ID to convert to another type of ID in the ID conversion module, which can be downloaded on the web page as a text file.

**Table 1.** Summary of input formats that RNAenrich supports.

Species	RNA type	ID type					
Human	miRNA	RNAcentral ID	miRBase ID (mature)	miRBase ID (stem-loop)	Official symbol	Gene ID	miRNA name
	lncRNA	RNAcentral ID	Official symbol	Gene ID	Ensembl ID	lncRNA name	
	circRNA	circRNA ID	circRNA name				
	snoRNA	RNAcentral ID	Official symbol	Gene ID	Ensembl ID	snoRNA name	
	mRNA	Gene ID	Gene name	Uniprot ID	Ensembl ID	RefSeq ID	
Mouse	miRNA	RNAcentral ID	miRBase ID (mature)	miRBase ID (stem-loop)	Official symbol	Gene ID	miRNA name
	lncRNA	RNAcentral ID	Official symbol	Gene ID	Ensembl ID	lncRNA name	
	circRNA	circRNA ID	circRNA name				
	snoRNA	RNAcentral ID	Official symbol	Gene ID	Ensembl ID	snoRNA name	
	piRNA	RNAcentral ID	piRNA name				
	mRNA	Gene ID	Official symbol	Uniprot ID	Ensembl ID	RefSeq ID	

## 3 Results

### 3.1 The realization of the enrichment in RNAenrich

Studies in ncRNAs are rapidly increasing. Uncovering the biological meaning of regulatory RNA molecules in living organisms is an important trend in the field of ncRNA research (Covarrubias *et al.* 2017, Zhang *et al.* 2018, Gregory 2019, Slack and Chinnaiyan 2019). Enrichment analysis is a well-used strategy to apply to explore molecular biological mechanisms (Delorey *et al.* 2021). A few emerging enrichment web servers focus on functional annotation and enrichment of ncRNAs (Li *et al.* 2018, Cardenas *et al.* 2020, Kern *et al.* 2020, Chen *et al.* 2021a); however, these servers have shown a series of limitations. First, the RNA types that these servers can analyze are not diverse, only covering miRNA (Li *et al.* 2018, Kern *et al.* 2020) or lncRNA (Chen *et al.* 2021a). Few tools can carry out enrichment analysis for other types of ncRNAs that also play crucial roles in living organisms, such as circRNA, piRNA, and snoRNA. Second, some existing tools (Li *et al.* 2018, Kern *et al.* 2020) conduct the enrichment analysis by directly matching each ncRNA with a term (pathway or disease) according to the literature, which may miss some key information, others (Cardenas *et al.* 2020) employ predicted data in built-in databases, which lead to low confidence in the enrichment results. Therefore, both types of mentioned tools cannot enable to conduct a comprehensive and accurate enrichment analysis for a list of ncRNAs.

**Figure 1.**
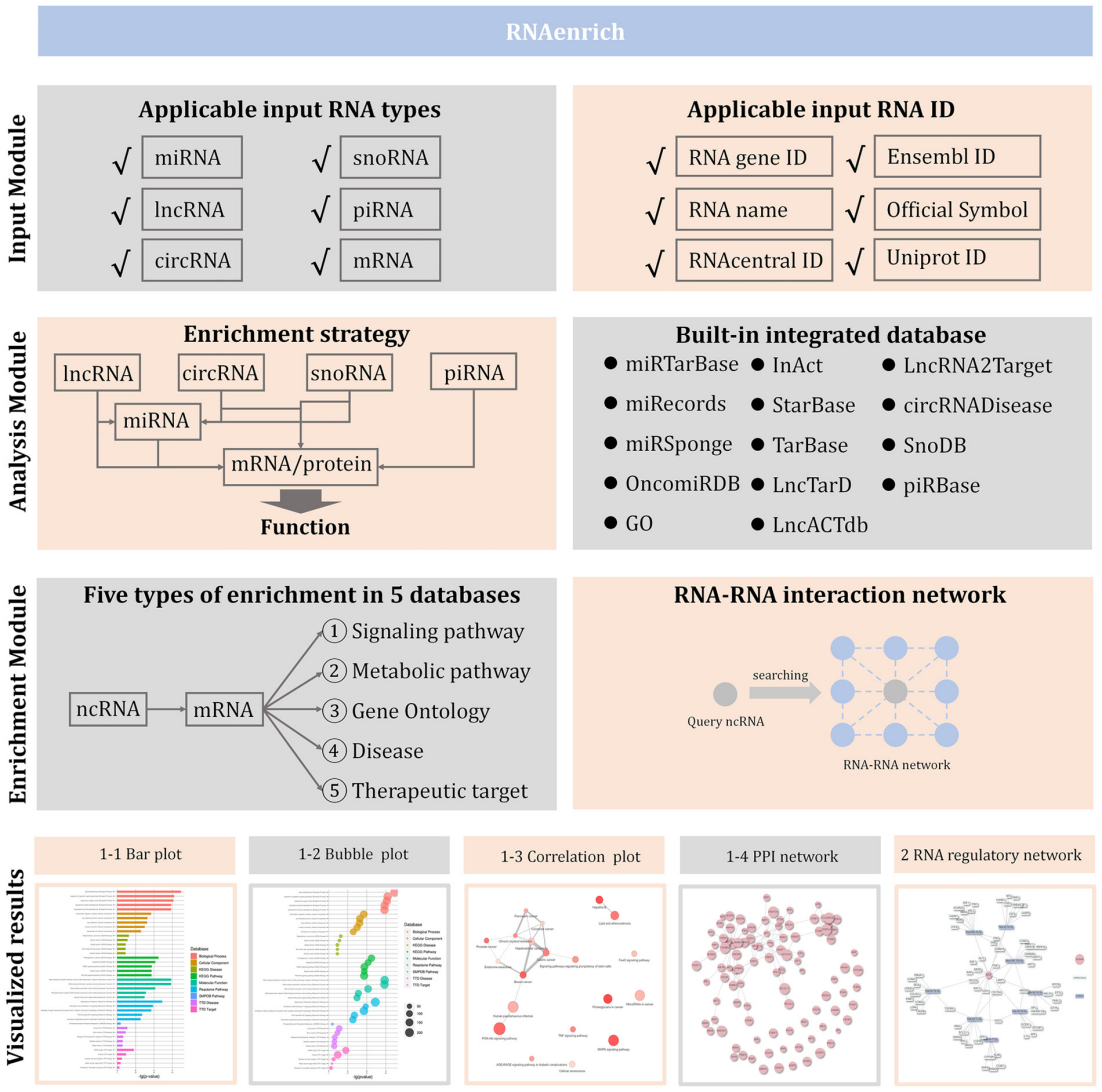
The workflow of RNAenrich. First, the users can input an RNA list; in this step, six types of RNA are optional. Then, the input RNA will be analyzed by built-in databases and a new enrichment strategy, matching a ncRNA with functional mRNA and then to a term. As a result, the tool will generate an enrichment analysis and an RNA–RNA interaction analysis, in which the former will be presented based on five different enrichments and the latter will profile a directional RNA–RNA regulatory network

In this study, many improvements have been made to overcome these limitations, including coverage of the most diverse types of ncRNA, enhanced enrichment accuracy, and introducing functional information about RNA–RNA interactions. Now, the web server can conduct a comprehensive analysis for any ncRNA set (Fig. 1). First, RNAenrich allows users to upload an RNA list, which can be a filtered RNA list from RNA sequencing or microarray, an interacting RNA list that is predicted by a computational approach, and so on. RNA types can be miRNA, lncRNA, circRNA, snoRNA, piRNA, or mRNA/gene, and the input format can be RNAcentral ID, Gene ID, Accession ID, Official Symbol, Ensembl ID, RNA name, miRbase ID (Mature miRNA), miRbase ID (Stem-loop miRNA), UniProt ID, or RefSeq ID (Table 1). Second, the input RNAs will be matched to their target mRNA or protein using the built-in database from 14 independent databases [miRTarBase (Huang *et al.* 2022), miRecords (Xiao *et al.* 2009), miRSponge (Wang *et al.* 2015), OncomiRDB (ling Chen *et al.* 2014), Gene Ontology (Huntley *et al.* 2016, Gene Ontology Consortium 2021), IntAct (Del Toro *et al.* 2022), StarBase (Fu *et al.* 2022), TarBase (Karagkouni *et al.* 2018), LncTarD (Zhao *et al.* 2020), LncACTdb (Wang *et al.* 2022b), LncRNA2Target (Cheng *et al.* 2019), circRNADisease (Zhao *et al.* 2018), SnoDB (Bouchard-Bourelle *et al.* 2020), and piRBase (Wang *et al.* 2022a)], which have experimentally validated ncRNA–target interactions of 1.87 million pairs covering all regulatory RNA types. Third, the mapped mRNAs will be enriched in five ways based on information from nine databases, namely, (i) signaling pathway, (ii) metabolic pathway, (iii) Gene Ontology, (iv) disease, and (v) therapeutic target; all of enrichment categories have attracted increasing attention in the field of RNA (Matsui and Corey 2017, Anastasiadou *et al.* 2018, Chen *et al.* 2018a, Zhang *et al.* 2019a, Zhu *et al.* 2019). Then, the enriched results will be presented as downloadable tables and visualized pictures, including bar plots, bubble plots, pathway correlation plots, and protein–protein interaction (PPI) networks. Specifically, the added PPI network will describe the regulatory molecular mechanism at the protein level in which the ncRNAs are involved. The network reveals PPIs that ncRNAs regulate via multiple signaling pathways. Finally, if users want to explore the RNA network that a query ncRNA regulates, RNA–RNA interaction module can provide an RNA–RNA regulatory network profile to show further detailed mechanisms.

### 3.2 The statistics of RNAenrich and current ncRNA enrichment server

Some existing tools employ a direct strategy, mapping an ncRNA to a term (a disease, a pathway, and so on) to generate an enrichment analysis (Table 2). Mechanically, as all ncRNAs execute their function depending on their associated coding RNAs or proteins, such a strategy may lead to the missing of target-associated functional information. Therefore, RNAenrich uses experiment-supported ncRNA–target interactions as built-in data to map their targets of query ncRNA list and capture the function of these targets by databases, such as KEGG and Reactome, which will generate a more comprehensive enrichment analysis relying on associated targets. Furthermore, these existing tools, such as TAM and MEAA, focus on the enrichment analysis of a single type of ncRNA, while RNAenrich includes five types of popular ncRNAs (miRNA, lncRNA, circRNA, snoRNA, and piRNA) and has sufficient coverage of ncRNA number in humans and mice (Table 3).

**Table 2.** Main content and function of RNAenrich and other representative web servers.

Server	RNA type	Evidence	Enriched strategy	Respective features	Reference
RNAenrich	Six types	Experiment-supported	ncRNA-mRNA-term	Pathway; metabolism; function; disease; therapeutic target; network	This study
TAM	miRNA	Experiment-supported	miRNA-term	Function; disease; transcription factor; classification; tissue and cell	(Li *et al.* 2018)
miEAA	miRNA	Experiment-supported	miRNA-term (most)	Function; disease; transcription factor; classification; tissue and cell; pathway; biomarker; localization	(Kern *et al.* 2020)
LncSEA	lncRNA	Experiment-supported	lncRNA-term	Function; disease; transcription factor; cancer; biomarker; regulation; drug	(Chen *et al.* 2021a)

**Table 3.** Comparison of RNAenrich and other representative web servers in coverage of RNA types and species.

Server	miRNA		lncRNA		circRNA		snoRNA		piRNA		mRNA	
	Human	Mouse	Human	Mouse	Human	Mouse	Human	Mouse	Human	Mouse	Human	Mouse
RNAenrich	**√**	**√**	**√**	**√**	**√**	**√**	**√**	**√**	**×**	**√**	**√**	**√**
TAM	**√**	**×**	**×**	**×**	**×**	**×**	**×**	**×**	**×**	**×**	**×**	**×**
miEAA	**√**	**√**	**×**	**×**	**×**	**×**	**×**	**×**	**×**	**×**	**×**	**×**
LncSEA	**×**	**×**	**√**	**×**	**×**	**×**	**×**	**×**	**×**	**×**	**×**	**×**

Taking advantage of these existing tools and adding popular trends to ncRNA research, the following five important aspects were simplified and summarized by RNAenrich to serve researchers (Table 2): (i) signaling pathway, to describe the protein-directed signaling conduction pathway that RNAs participate in, such as lncRNA AK023391 for the PI3K/Akt signaling pathway (Huang *et al.* 2017) and circIKBKB for the NF-κB pathway (Xu *et al.* 2021); (ii) metabolic pathway, to describe the metabolite-based pathway or reaction that RNAs regulate, such as lncRNA HISLA for aerobic glycolysis (Chen *et al.* 2019a) and miR-147b for the TCA cycle (Zhang *et al.* 2019b); (iii) Gene Ontology, to describe the functional role of ncRNAs and their contribution to biological processes and location in the cell, such as lncRNA SLERT for transcription (Wu *et al.* 2021) and lncRNA LETN for nucleolar structure (Wang *et al.* 2021c); (iv) disease, to describe involvement of ncRNA in the occurrence or development of disease or complications, such as miRNA-342-3p for hepatocellular carcinoma (Komoll *et al.* 2021) and LINC01123 for non-small cell lung cancer (Hua *et al.* 2019); and (v) therapeutic target, such as miRNA let-7 for immunotherapy (Gilles and Slack 2018) and lncRNA H19 for therapeutic target of pancreatic cancer (Wang *et al.* 2020). Each of these aspects has contributed to a bulk of publications, to reveal the physiological and pathophysiological mechanism, by exploration of ncRNA associations.

### 3.3 Comparing RNAenrich with existing tools based on COVID-19 data

To date, two online tools have already been developed for conducting an enrichment analysis for miRNAs, including TAM (Li *et al.* 2018) and miEAA (Kern *et al.* 2020). TAM is a very popular tool for enrichment analysis of a list of miRNAs, and has been applied to the research on molecular mechanism and signaling pathway of miRNAs in diverse diseases (Soleimani Zakeri *et al.* 2020). Compared with the TAM, miEAA is a more comprehensive web server for miRNA enrichment and annotation, which has made many improvements, such as providing the most coverage of miRNAs, enhancing functional diversity, and integrating multiple species (Kern *et al.* 2020). As reported, the significance and abundance of enrichment results are key indicators of enrichment analysis (Yang *et al.* 2021). In other words, the more significant and abundant results a server can enrich, the more valuable information it can provide, facilitating further discovery and analysis of biological molecular mechanisms for ncRNAs (Yang *et al.* 2021). Therefore, in this study, we chose miEAA to compare with RNAenrich in term of the significance and abundance of enrichment results.

We use a miRNA list from Khan’s study (Khan *et al.* 2020) as the test data, in which 106 human miRNAs are predicted to interact with SARS-CoV-2 genomic RNA. This study concluded that these miRNAs could regulate immune-signaling pathways and viral infection processes. In other words, the enrichment report of these miRNAs should be related to immune-signaling pathways, inflammation-related pathways, and virus-related diseases. Therefore, we analyzed the test list with RNAenrich and miEAA and compared the enrichment results. For the involved-signaling pathway, we summarized the pathways directly related to COVID-19. According to the authoritative studies by Wiersinga *et al.* (2020) and Blanco-Melo *et al.* (2020), COVID-19 presents as a viral infection and replication in the early stage, and as excessive inflammatory responses and immunological stress (typical as interleukin-induced pathways) in the late stage of infection. Therefore, we filtered virus infection-, interleukin-, and immune system-related pathways in the enrichment results from both tools.

**Figure 2.**
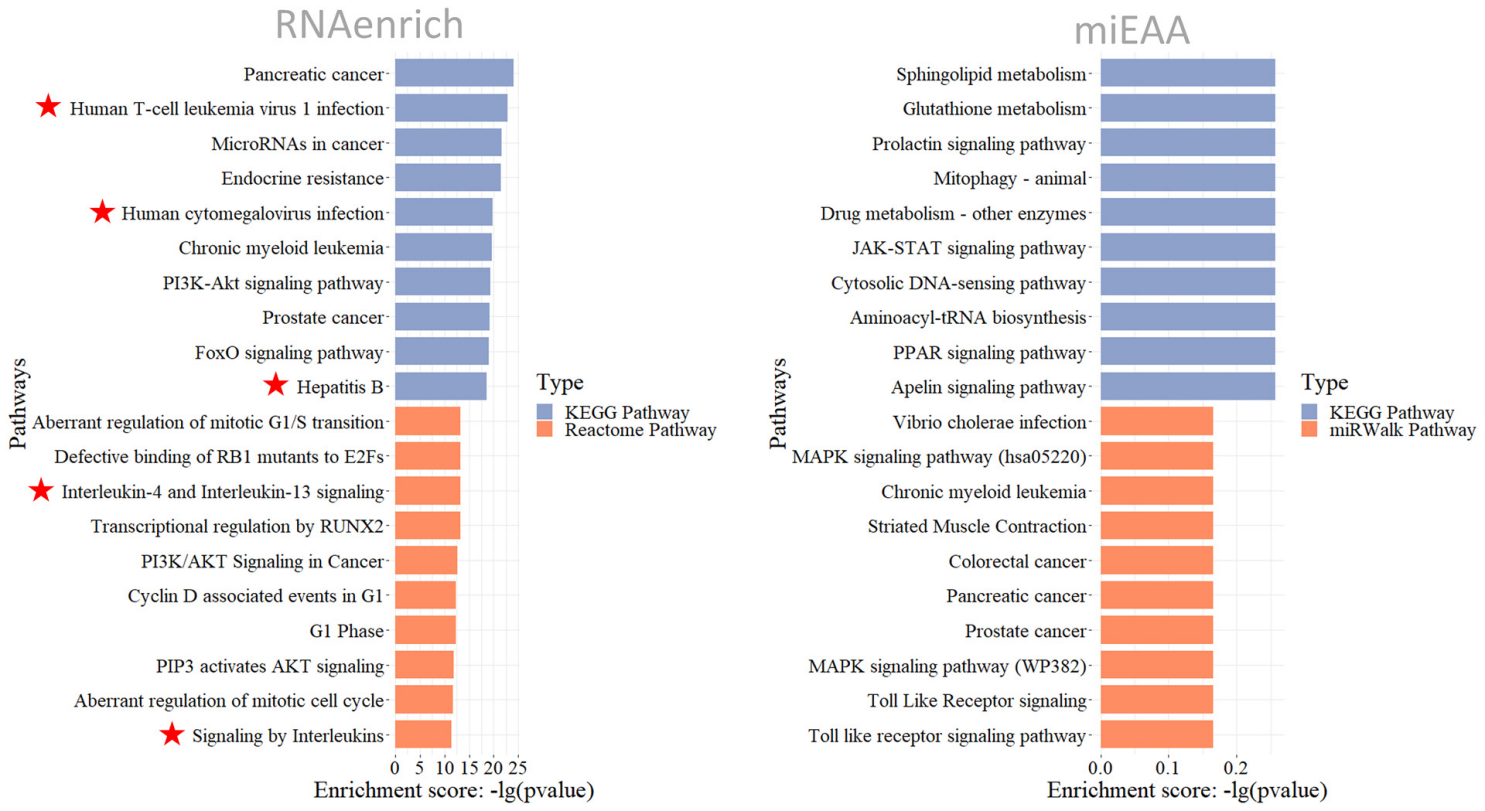
Pathway enrichment results (top 10) of RNAenrich (left) and miEAA (right) for a COVID-19-related miRNA list as input. Pathway enrichment patterns included KEGG and Reactome by RNAenrich and KEGG and miRWalk by miEAA

Interestingly, a series of COVID-19 related pathways were significantly enriched by RNAenrich (shown in Fig. 2 and Table 4), e.g. human interleukin-4 and interleukin-13 signaling (*P* = 5.74E-14) (*P* refers to *P*.adjust), interleukin-6 signaling (*P* = 8.64E-04), and so on (shown in Table 4 and Supplementary Tables S1 and S2). However, miEAA enrichment identified some unrelated pathways (shown in Fig. 2 and Supplementary Tables S3 and S4). For disease enrichment, RNAenrich captured some SARS-CoV-2-related or similar diseases (shown in Table 4), including COVID-19 (*P* = 3.49E-02), and lupus erythematosus (a type of immune system disease) (*P* = 8.28E-03); however, miEAA failed to significant diseases (shown in Supplementary Table S5), with some non-significant related terms. As shown in Table 4, a series of COVID-19-related diseases and signaling pathways were significantly enriched in RNAenrich but not in miEAA (shown in Supplementary Table S6). Overall, RNAenrich has illustrated its enhanced performance in enriching the COVID-19-related miRNA list compared with miEAA.

**Table 4.** SARS-CoV-2-related pathways and diseases based on enrichment results from RNAenrich with a significant difference (*P*.adjust < 0.05).

Description	Rate	*P*-value	*P*.adjust
Human T-cell leukemia virus 1 infection	41/222	3.07E−24	2.53E−22
Human cytomegalovirus infection	39/225	5.20E−22	2.14E−20
Hepatitis B	32/162	6.23E−20	1.40E−18
Human papillomavirus infection	42/331	2.33E−18	3.39E−17
Epstein–Barr virus infection	32/202	6.02E−17	6.76E−16
Kaposi sarcoma-associated herpesvirus infection	30/194	1.23E−15	1.21E−14
Hepatitis C	27/157	2.40E−15	2.20E−14
Interleukin-4 and interleukin-13 signaling	23/108	1.91E−16	5.74E−14
Signaling by interleukins	41/462	3.83E−14	3.68E−12
Human immunodeficiency virus 1 infection	20/212	5.11E−07	1.58E−06
Interleukin-6 signaling	4/11	7.61E−05	8.64E−04
Interleukin-10 signaling	7/47	8.39E−05	9.41E−04
Interleukin-3, interleukin-5, and GM-CSF signaling	7/48	9.63E−05	1.04E−03
Interleukin-6 family signaling	5/24	1.75E−04	1.63E−03
Interleukin-15 signaling	4/14	2.19E−04	1.95E−03
Coronavirus disease—COVID-19	15/232	9.80E−04	2.03E−03
Interleukin receptor SHC signaling	5/27	3.14E−04	2.60E−03
Interleukin-2 family signaling	6/44	4.47E−04	3.53E−03
Interleukin-7 signaling	5/36	1.24E−03	8.16E−03
Lupus erythematosus	9/45	8.57E−04	8.28E−03
Cutaneous lupus erythematosus	4/11	2.66E−03	1.93E−02
COVID-19	10/70	6.26E−03	3.49E−02

### 3.4 Visualization of enrichment results in RNAenrich

**Figure 3.**
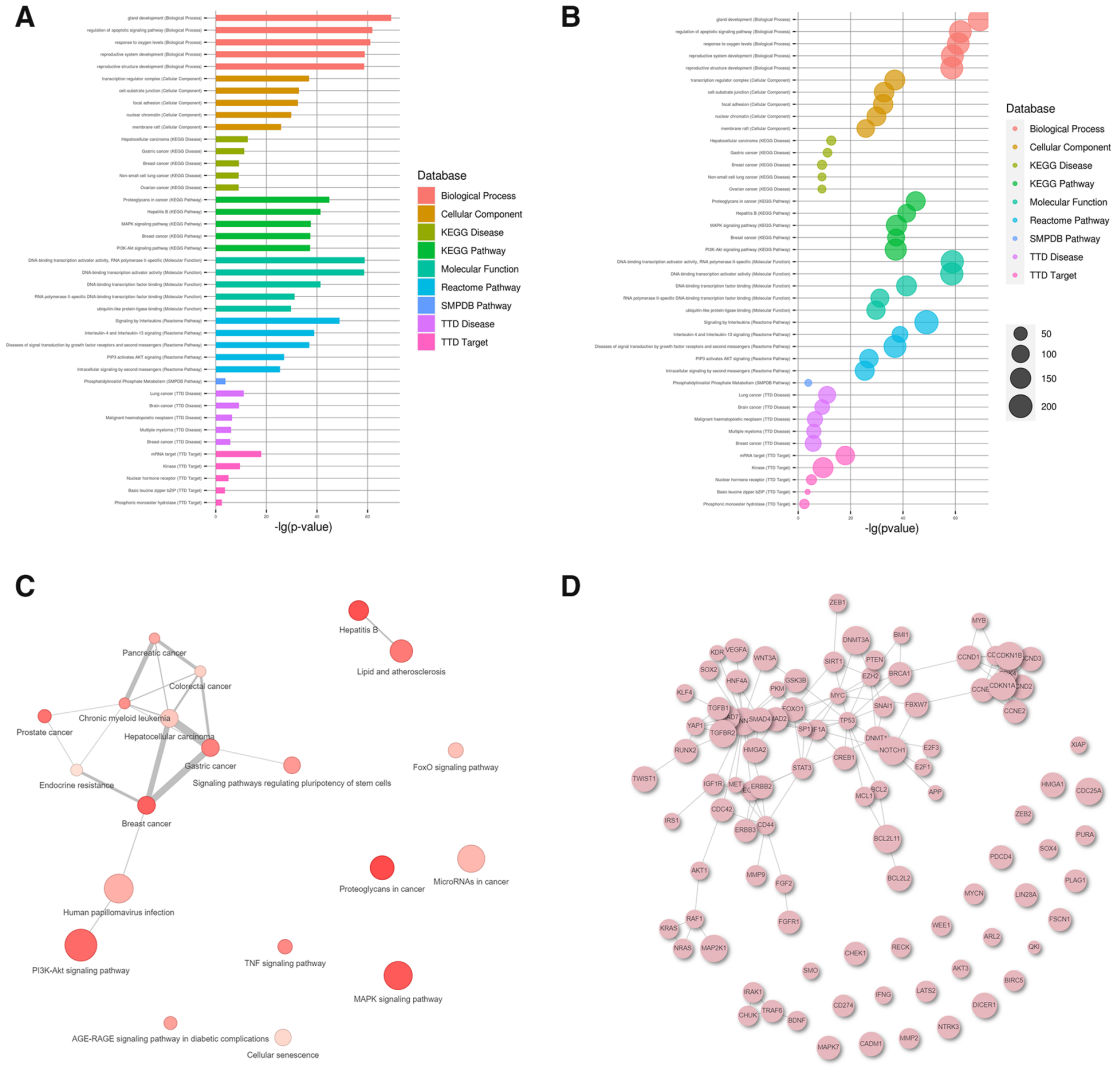
Visualization of the enrichment results by RNAenrich. Bar (A) and bubble (B) plots of enrichment results in five databases. (C) The correlation plot of enrichment results in a single database. (D) The PPI network of proteins that ncRNAs regulate is shown

Currently, two functions can be carried out by RNAenrich for an ncRNA list, RNA functional enrichment and RNA–RNA interaction network analysis. According to the RNAenrich procedure, users first input the RNA list and choose some related options and then obtain the result page after 5–60 s (the waiting time depends on the number of selected databases). The result page first shows the following information: (i) an enrichment analysis report with term name, database, link ID, *P*-value, *P*.adjust, and so on, which is presented as a table and can be downloaded as CSV file; (ii) visualized plots of the enrichment results (bar plot, bubble plot, and correlation plot for selectable one and more databases) shown in Fig. 3A–C; and (iii) a PPI network plot (shown in Fig. 3D) to describe which PPI pairs are regulated by these ncRNAs, and these nodes with more neighbors may be key proteins that ncRNA can regulate.

**Figure 4.**
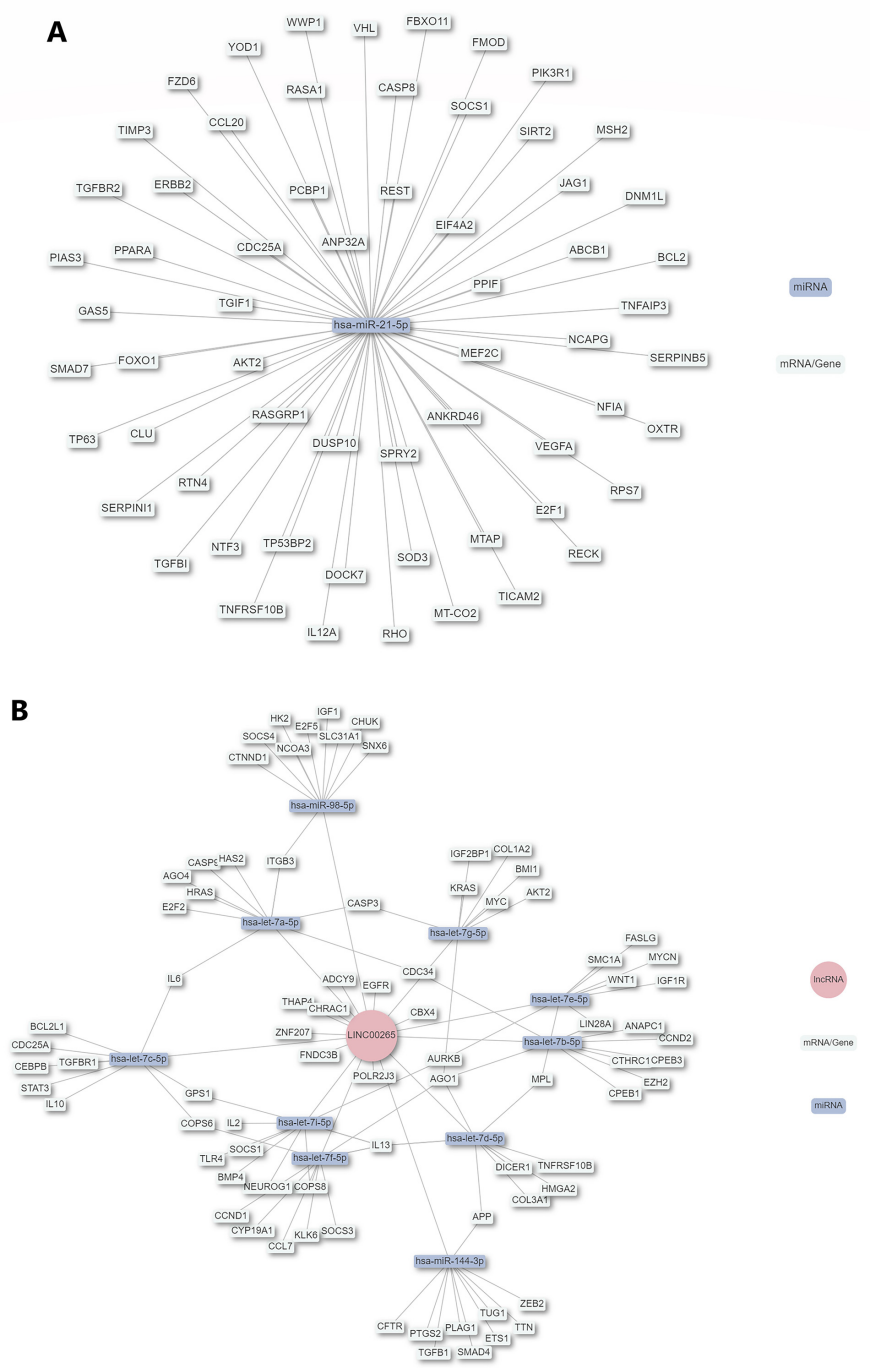
Visualization of the RNA–RNA interaction network. The regulatory networks that miRNAs (A) and lncRNAs (B) direct. (**A**) The miRNA-central network consists of a series of miRNA–mRNA regulations, and (**B**) the lncRNA-central network consists of diverse regulations, such as lncRNA–miRNA, miRNA–mRNA, lncRNA–mRNA, and lncRNA–protein.

As the regulation among different RNAs is of great concern, e.g. miRNA–mRNA (Yoo *et al.* 2021b), lncRNA–miRNA–mRNA (Wasson *et al.* 2021), and circRNA–miRNA–mRNA (Neumann *et al.* 2018), our server also provides RNA–RNA interaction information in a downloadable table and picture to describe the regulatory network that a ncRNA is involved in. As shown in Fig. 4A, the miRNA regulatory network is shown in this way because its regulatory mode is simple. For lncRNAs, the mechanism of action is more diverse, such as binding to DNA promoters, miRNAs, mRNAs, and proteins, therefore, the regulatory network of lncRNA is complicated and crosslinked (shown in Fig. 4B), which may provide more regulatory information to uncover molecular mechanisms in diseases for users.

## 4 Conclusions

In this study, an online tool, RNAenrich, was developed to enable comprehensive enrichment analysis of ncRNAs. This tool is unique in that (i) it can be used for enrichment analysis for various RNA types, such as circRNAs, snoRNAs, piRNAs, miRNAs, lncRNAs, and mRNAs; (ii) the analysis can be extended by introducing millions of experimentally validated RNA–target interactions as a built-in database; and (iii) the results provide a comprehensive interacting network among various ncRNAs and targets to facilitate the mechanistic study of ncRNA function. Interest in the exploration of ncRNAs is gradually extending and transforming from interest in miRNAs and lncRNAs into interest in newly identified ncRNAs, such as circRNAs, piRNAs, and snoRNAs. RNAenrich now enables enrichment analysis of all RNA types, which will be beneficial for researchers from different ncRNA fields. Studies on ncRNAs are ongoing and expanding, and RNAenrich will also be improving to better serve the field of ncRNA research.

## Supplementary Material

btad421_Supplementary_Data

## Data Availability

The data underlying this article are available in the article and in its online supplementary material.
